# BEscreen: a versatile toolkit to design base editing libraries

**DOI:** 10.1093/nar/gkaf406

**Published:** 2025-05-19

**Authors:** Philipp G Schneider, Shuang Liu, Lars Bullinger, Benjamin N Ostendorf

**Affiliations:** Department of Hematology, Oncology, and Tumor Immunology, Charité-Universitätsmedizin Berlin, 13353 Berlin, Germany; Berlin Institute for Medical Systems Biology (BIMSB), Max Delbrück Center for Molecular Medicine, 10115 Berlin, Germany; Department of Hematology, Oncology, and Tumor Immunology, Charité-Universitätsmedizin Berlin, 13353 Berlin, Germany; Berlin Institute for Medical Systems Biology (BIMSB), Max Delbrück Center for Molecular Medicine, 10115 Berlin, Germany; Department of Hematology, Oncology, and Tumor Immunology, Charité-Universitätsmedizin Berlin, 13353 Berlin, Germany; German Cancer Consortium (DKTK), Partner Site Berlin, and German Cancer Research Center (DKFZ), 69120 Heidelberg, Germany; National Center for Tumor Diseases (NCT), Partner Site, 13353 Berlin, Germany; Department of Hematology, Oncology, and Tumor Immunology, Charité-Universitätsmedizin Berlin, 13353 Berlin, Germany; Berlin Institute for Medical Systems Biology (BIMSB), Max Delbrück Center for Molecular Medicine, 10115 Berlin, Germany; German Cancer Consortium (DKTK), Partner Site Berlin, and German Cancer Research Center (DKFZ), 69120 Heidelberg, Germany; Berlin Institute of Health, 10178 Berlin, Germany

## Abstract

Base editing enables the high-throughput screening of genetic variants for phenotypic effects. Base editing screens require the design of single guide RNA (sgRNA) libraries to enable either gene- or variant-centric approaches. While computational tools supporting the design of sgRNAs exist, no solution offers versatile and scalable library design enabling all major use cases. Here, we introduce BEscreen, a comprehensive base editing guide design tool provided as a web server (bescreen.ostendorflab.org) and as a command line tool. BEscreen provides variant-, gene-, and region-centric modes to accommodate various screening approaches. The variant mode accepts genomic coordinates, amino acid changes, or rsIDs as input. The gene mode designs near-saturation libraries covering the entire coding sequence of given genes or transcripts, and the region mode designs all possible guides for given genomic regions. BEscreen enables selection of guides by biological consequence, it features comprehensive customization of base editor characteristics, and it offers optional annotation using Ensembl’s Variant Effect Predictor. In sum, BEscreen is a highly versatile tool to design base editing screens for a wide range of use cases with seamless scalability from individual variants to large, near-saturation libraries.

## Graphical Abstract

**Graphical Abstract:**
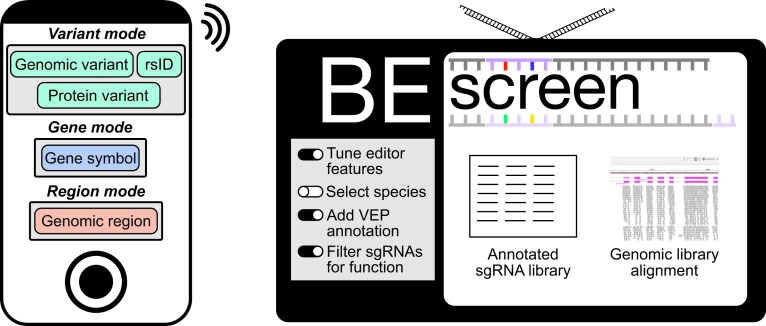

## Introduction

Genetic variation is a central driver of phenotypic diversity, contributing to both physiological variability and susceptibility to a wide range of diseases [1]. The advent of next-generation sequencing has allowed for determining the association between many genetic variants with specific phenotypes in large human cohorts [2]. However, the assessment of which variants causally drive a given phenotype has remained a central challenge in the field [3].

Base editors (BEs) catalyze the conversion of nucleotides; cytosine base editors (CBEs) convert a C•G to a T•A base pair, while adenine base editors (ABEs) convert an A•T to a G•C base pair. ABEs and CBEs consist of a deaminase coupled to a catalytically impaired version of Cas9. This combination enables the use of single guide RNAs (sgRNAs) to direct the deaminase to specific sites in the genome, thus enabling the modification of specific genomic loci [4–6]. More recently, BEs have been explored that catalyze additional nucleotide modifications [7, 8]. By transducing a pool of base editor-expressing cells with a library of sgRNAs to introduce multiple variants in parallel, base editing can be leveraged for the high-throughput assessment of variants for causal effects at scale [9, 10]. Recent studies have demonstrated the potential of such BE screens, leading to the identification of single nucleotide polymorphisms (SNPs) that modulate diverse phenotypes, including T cell function [11, 12], drug responses [13], and signaling [14, 15].

BE screens require computational tools to facilitate the design of sgRNA libraries. While several tools that aid in the design of BE sgRNAs exist [16–21], no tool provides broad and flexible functionality to address all major use cases for BE screens. These use cases range from interrogating specific, pre-defined variants to assessing all possible edits in given genes, transcripts, or otherwise defined genomic regions. In addition, over the last few years, a range of different BEs have been pioneered that exhibit distinct characteristics, including different editing windows, different requirements for the presence of protospacer adjacent motifs (PAMs), and others, requiring flexible options in an sgRNA design tool [5, 6, 22–25].

Here, we introduce BEscreen, a versatile toolkit that enables the design of BE sgRNA libraries for a broad range of use cases (Supplementary Fig. S1). BEscreen accepts different inputs, including genomic coordinates, protein mutations, genes, transcripts, and genomic regions. It thus aids in the design and functional annotation of sgRNAs for both variant- and gene-centric applications. Its capacity to filter sgRNAs for given biological consequences makes it uniquely suitable to design screening libraries with defined positive and negative controls. BEscreen also provides unprecedented flexibility in terms of defining PAM requirements and other base editor characteristics.

## Materials and methods

### BEscreen offers three modes focused on variants, genes, and genomic regions

**Figure 1.**
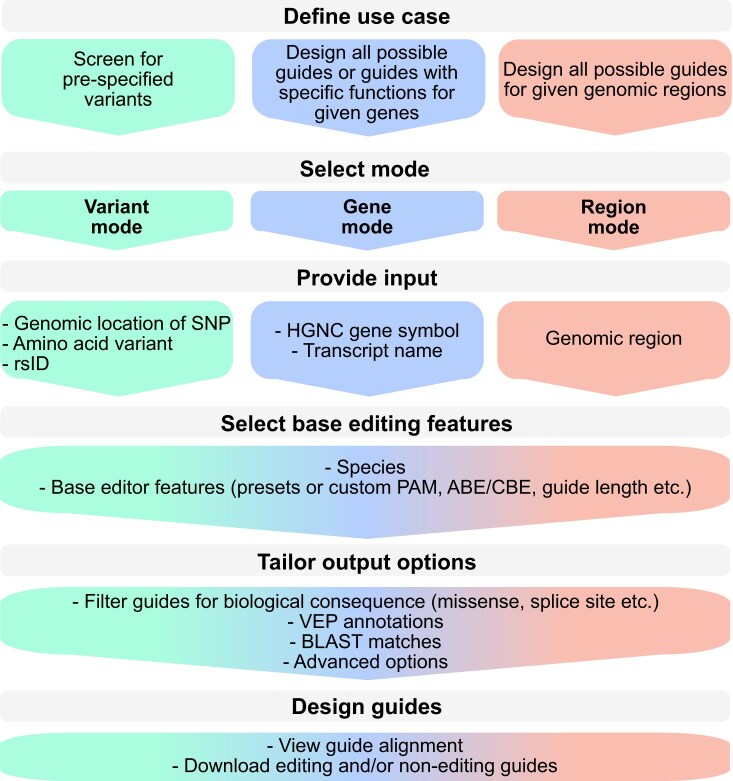
Use cases of BEscreen. Flowchart illustrating the use cases enabled by BEscreen’s three modes (variant-, gene-, and region-based). BEscreen can be used to identify sgRNAs for specific variants (nucleotide- or amino acid-defined) as well as for genes and genomic regions. ABE, adenine base editor; CBE, cytosine base editor; HGNC, HUGO Gene Nomenclature Committee; PAM, protospacer adjacent motif; SNP, single nucleotide polymorphism; VEP, Variant Effect Predictor.

BEscreen offers three different modes to design base editing guides for pre-defined variants, for coding sequences (CDS) of specific genes and transcripts, and for given genomic regions. Input can be provided manually or as a CSV file. An overview of the workflow using BEscreen is outlined in Fig. 1.

#### Variant-centric mode

BEscreen's variant mode identifies any possible sgRNA that mediates editing of a pre-defined nucleotide or amino acid variant. Nucleotide variants are accepted as a concatenated string composed of the genomic coordinates and reference and alternative bases. For example, “12_6537866_C_T” indicates a variant with a thymidine instead of a cytosine on chromosome 12 at position 6537866. Alternatively, nucleotide variants can be provided using their rsID from dbSNP (e.g. “rs1062436”).

Amino acid mutations can be provided as gene or transcript name concatenated with an amino acid substitution (e.g. “GAPDH-L270F” for a leucine to phenylalanine substitution at position 207 in the default *GAPDH* transcript or “GAPDH-201-L270F” for the same variant in transcript 201 of *GAPDH*). BEscreen assesses whether the desired mutation can be introduced by editing a single base in one of the three bases making up the codon. If so, it will convert this edit to genomic coordinates and design guides accordingly.

#### Gene-centric mode

BEscreen's gene mode identifies any possible sgRNA in the CDS of given genes or transcripts to generate near-saturation BE libraries. The gene mode accepts HUGO Gene Nomenclature Committee (HGNC) gene names (e.g. “GAPDH”) as input with the option to specify a given transcript (e.g. “GAPDH-201” for *GAPDH* transcript 201). It then extracts the nucleotide sequence of all CDS regions in the specified gene(s) or transcript(s) and slides through this sequence searching for combinations of PAM sites and editable bases on both strands to identify and annotate guide sequences.

#### Region-centric mode

BEscreen's region mode accepts a genomic region as input (e.g. “12:6536490–6537490” for region 6536490–6537490 on chromosome 12). BEscreen extracts the nucleotide sequence of indicated regions and slides through the sequence searching for combinations of PAM sites and editable bases on both strands to identify all possible sgRNAs.

### BEscreen is highly customizable

BEscreen offers extensive customization options for all three modes. Notably, BEscreen enables full customization of PAM site requirements and base editor characteristics, including adjusting the length of the desired guides and the position of the editing window. BEs are expected to exhibit high editing efficiency in their individually determined editing window, but edits may occur outside of this window, albeit at lower efficiency [20]. To account for this, users can define a safety region to annotate guides with possible off-target edits in regions of minor editing efficiency adjacent to the main editing window. For convenience, all parameters can be set using base editor presets [18, 20, 21, 23, 26].

In addition to customization options for input and base editor characteristics, BEscreen enables tailoring of the output. Importantly, as a part of this, BEscreen provides functionality to identify guides with given biological consequences. This feature enables convenient identification of screen controls, such as synonymously editing guides as negative controls and splice site disrupting or nonsense variant-mediating guides as positive controls.

### BEscreen web server implementation

The BEscreen web server (bescreen.ostendorflab.org) is a Shiny for Python-based web app (v1.2.1; Python v3.12.3). The main third-party modules used are Polars (v1.16.0; github.com/pola-rs/polars) for data processing, pyfaidx (v0.8.1.3; github.com/mdshw5/pyfaidx) to extract genomic sequences from a FASTA reference genome, and PyRanges (v0.1.2; [27]) to parse a GTF annotation file. Optional annotation is based on Ensembl’s Variant Effect Predictor (VEP; v113.3; [28]) and NCBI’s BLAST (v2.16.0; [29]). igv.js (v3.1.3; [30]) is used with BAM files created by pysam (v0.22.1; github.com/pysam-developers/pysam) to display the alignment of sgRNAs to the reference genome. BEscreen provides the Ensembl reference genomes for human (GRCh38, Release 112) and mouse (GRCm39, Release 112) and is running on Ubuntu (24.04.1 LTS) and Apache2 (version 2.4.58). A command-line version is available at github.com/ostendorflab/bescreen.

## Results

### BEscreen provides rich output

BEscreen's main output is a table providing detailed information of the identified guides, including the introduced variant(s), base change, guide sequence with and without PAM site, sequence of the editing window (and, if set, for the safety region), the genomic location, and the strand of guide alignment. In addition, the table details how many edits take place within the editing window, and the distance of these edits to the center of the editing window, where editing is most efficient. For the variant and gene modes, BEscreen annotates identified guides with the affected gene, transcript, and exon. In addition, BEscreen reports affected codons and their translation product pre- and post-editing with their positions. In the gene and region modes, output includes an additional table listing non-editing guides (sgRNAs predicted to introduce no edits).

Optionally, the output of BEscreen can be annotated using Ensembl’s VEP [28]. To identify additional binding sites of given guides, BEscreen can identify matches of the guide sequence in the reference genome using NCBI’s BLAST [29]. In addition to the tabular output, the BEscreen web server displays genomic alignment of the identified sgRNAs in an embedded version of the Integrative Genome Viewer (igv.js) [30].

### Case study

To illustrate BEscreen's functionality in practice, we designed sgRNAs for variants in the *APOE* gene using the most commonly used ABE and CBE BEs. *APOE* variants are the largest monogenetic risk modifiers of Alzheimer's disease and have more recently been implicated in modulating additional phenotypes ranging from cardiovascular disease to tumor and infection immunity [31–35].

**Figure 2.**
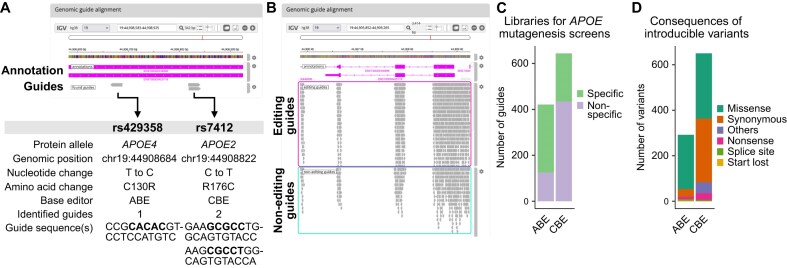
Case study: design of individual guides and sgRNA libraries for variants in the *APOE* gene. (**A**) Overview of the output of BEscreen used in variant mode to identify guides introducing the clinically significant rs429358 and rs7412 variants in the *APOE* gene. (**B**) Genomic alignment of a subset of the guides identified by BEscreen's gene mode to identify all possible editing and non-editing guides in the *APOE* coding sequence. (**C**) Number of editing guides identified for ABE (adenine base editor) and CBE (cytosine base editor) as shown in panel (B), indicating whether guides are predicted to be specific (mediate only one edit) or not. (**D**) Overview of the consequences mediated by editing guides shown in panel (B).

Three highly prevalent variants of *APOE* exist, termed *APOE2*, *APOE3*, and *APOE4*, which are defined by two SNPs. The first SNP (rs429358) is a T to C substitution on chromosome 19 at position 44908684 leading to a change of a TGC codon to CGC, resulting in a cysteine to arginine substitution (APOE^C130R^). The second SNP (rs7412) corresponds to a C to T substitution at position 44908822 (APOE^R176C^). The most common *APOE3* variant is defined by the rs429358(T)/rs7412(C) genotype, while the *APOE4* and *APOE2* variants correspond to rs429358(C)/rs7412(C) and rs429358(T)/rs7412(T) genotypes, respectively. We used BEscreen's variant mode to identify guides that mediate the introduction of these mutations in the reference *APOE3* background (Fig. 2A). BEscreen identified one guide suitable for the rs429358 edit. Notably, inspection of the guide's characteristics in the tabular output revealed that this guide introduces a second edit changing the upstream GTG codon to GCG, resulting in an additional missense mutation. For the rs7412 SNP, BEscreen identified two suitable guides mediating the desired APOE^R176C^ mutation. By entering variants at the protein instead of nucleotide level (i.e. APOE-C130R instead of rs429358), BEscreen searches for whether additional edits in any of the other two bases making up the affected codon achieve the intended protein consequences, which here was not the case.

The implications of other, less common *APOE* variants remain largely unexplored, rendering their systematic interrogation intriguing [36]. To design an sgRNA library introducing all possible edits into *APOE*, we next leveraged BEscreen's gene mode (Fig. 2B). Using “APOE” as input, BEscreen designed 1065 editing guides, 506 of which mediated only one edit and were thus annotated as specific (Fig. 2C). As detailed in BEscreen's output, the consequences mediated by all edits included 526 missense, 313 synonymous, 11 splice site, 48 nonsense, 2 start lost, and 2 stop lost edits (Fig. 2D). In addition, 402 binding but non-editing guides were identified. These non-editing guides and guides conferring synonymous edits are valuable as negative controls in a BE screen, while guides mediating splice site disruptions or nonsense mutations may serve as positive controls. Thus, BEscreen designed a complete library for evaluating known and unknown variants in the *APOE* gene with minimal and convenient input requirements.

Finally, we used the genomic region spanning the *APOE* gene as input for BEscreen's region mode (“19:44905791–44909393”), resulting in 3150 and 1247 editing and non-editing guides, respectively. Notably, these guides cover the entire gene region, including introns and 5′ and 3′ untranslated regions (UTRs) in contrast to the output of the gene mode.

## Discussion

CRISPR-Cas9-coupled base editing has enabled the rapid adoption of functional screens to interrogate the causal impact of genetic variants at scale [9, 10]. BEscreen is a versatile toolkit to design sgRNAs from small-scale experiments focusing on pre-specified single nucleotide and amino acid variants to large-scale near-saturation screens of genes and transcripts or genomic regions. BEscreen is uniquely suited to design positive and negative controls by allowing for sgRNA selection by biological consequence, including synonymous or non-editing (for negative controls) and nonsense or splice site mutations (for positive controls). BEscreen features three modes that enable BE screens focused on variants, genes, or genomic regions. Future iterations of BEscreen may include more refined quality control metrics as biological characteristics of specific BEs become better characterized and will support additional nucleotide changes as novel nucleotide editing enzymes are discovered or engineered. In sum, BEscreen provides a comprehensive tool for the design of sgRNA libraries for base editing screens.

## Supplementary Material

gkaf406_Supplemental_File

## Data Availability

The BEscreen web server is available at bescreen.ostendorflab.org. This website is free and open to all users and there is no login requirement. A command line tool for local installation is available at github.com/ostendorflab/bescreen and at doi.org/10.6084/m9.figshare.28616528.
