# Supplementary material for: BEscreen: a versatile toolkit to design base editing libraries
Source: Nucleic Acids Res. 2025 May 19;53(W1):W68–72. doi: 10.1093/nar/gkaf406 (PMC12230696; doi:10.1093/nar/gkaf406)
Supplement: gkaf406_Supplemental_File [file gkaf406_supplemental_file.pdf]

BEscreen: a versatile toolkit to design base editing libraries

Philipp G. Schneider[1,2], Shuang Liu[1,2], Lars Bullinger[1,3,4], Benjamin N. Ostendorf[1,2,3,5,*]

[1] Department of Hematology, Oncology, and Tumor Immunology, Charité-Universitätsmedizin Berlin, Berlin, Germany
[2] Berlin Institute for Medical Systems Biology (BIMSB), Max Delbrück Center for Molecular Medicine, Berlin, Germany
[3] German Cancer Consortium (DKTK), Partner Site Berlin, and German Cancer Research Center (DKFZ), Heidelberg, Germany
[4] National Center for Tumor Diseases (NCT), Partner Site, Berlin, Germany
[5] Berlin Institute of Health, Berlin, Germany

* Correspondence: benjamin.ostendorf@charite.de

SUPPLEMENTARY DATA

Supplementary Figure 1

	BEscreen [1]	PnB-Designer [2]	SNP-CRISPR [3]	beditor [4]	BE-Designer [5]	base-editor-design-tool [6]	BE-target [7]
**Availability**							
Web server	✓	✓	✓	✗	✓	✗	✓
Command line interface	✓	✓	✓	✓	✗	✓	✗
No login/E-Mail required	✓	✓	✗	✓	✓	✓	✓
**Guide customization**							
Guide length	✓	✗	✗	✗	✓	✓	✓
Fully customizable PAM	✓	✗	✗	✗	✗	✗	✓
Editing window	✓	✗	✗	✗	✓	✓	✗
**Input options**							
Manual input	✓	✓	✗	✗	✓	✗	✓
File input	✓	✓	✓	✓	✓	✓	✗
Genomic location	✓	✓	✓	✓	✗	✗	✗
Amino acid change	✓	✗	✗	✓	✗	✗	✗
dbSNP rsID	✓	✗	✗	✗	✗	✗	✗
Genomic regions	✓	✗	✗	✓	✓†	✓	✓‡
Gene (CDS)	✓	✗	✗	✗	✗	✓	✗
**Output options**							
Filter by biol. consequence	✓	✗	✗	✗	✗	✗	✗
Splice site recognition	✓	✗	✗	✗	✗	✗	✗
Vis. of genomic alignment	✓	✗	✗	✗	✗	✗	✗
Results download	✓	✓	✗*	✗	✓	✗	✓

*not functional at time of access
†(≤1 kbp) ‡ (≤10 kbp)

Links:
[1] bescreen.charite.de
[2] fgcz-shiny.uzh.ch/
[3] flyrnai.org/tools/snp_crispr/web/
[4] pypi.org/project/beditor/
[5] rgenome.net/be-designer/
[6] github.com/mhegde/base-editor-design-tool
[7] skl.scau.edu.cn/targetdesign/

Supplementary Figure 1: Comparison of BEscreen's features with alternative tools.
